# True Posterior Communicating Artery Aneurysm Associated With a Posterior Communicating Segment Aneurysm: A Case Report

**DOI:** 10.7759/cureus.69186

**Published:** 2024-09-11

**Authors:** Ambar Elizabeth Riley Moguel, Alejandro Serrano-Rubio, José Alfredo González Soto, Janeth N. Nuñez-Lupaca, Edgar Nathal

**Affiliations:** 1 Neurosurgery, Instituto de Seguridad y Servicios Sociales de los Trabajadores del Estado (ISSSTE), Mexico City, MEX; 2 Vascular Neurosurgery, Instituto Nacional de Neurología y Neurocirugía Manuel Velasco Suárez, Mexico City, MEX; 3 General Practice, Universidad Autónoma Metropolitana, Mexico City, MEX; 4 Neurosurgery, Universidad Nacional Jorge Basadre Grohmann, Tacna, PER; 5 Neurosurgery, Instituto Nacional de Neurología y Neurocirugía Manuel Velasco Suárez, Mexico City, MEX

**Keywords:** aneurysms, anterior ressection of uncus, microsurgical aneurysm clipping, posterior communicant artery (pcom), true posterior communicant artery aneurysms

## Abstract

"True" posterior communicating artery (PComA) aneurysms are one of the rarest of all intracranial aneurysms. Diagnosis of this kind of aneurysm and treatment continue to be challenging for neurosurgeons because of the surrounding structures and their importance. Concomitant vascular anomalies, like dissections or hypoplasia, are frequently found with these aneurysms. Multiple aneurysms in the posterior communicating segment are even rarer than true PComA aneurysms alone. To achieve a safe clipping in patients with multiple aneurysms in this location, some coadjuvant maneuvers may be performed, like resection of the anterior part of the uncus. By presenting this case, we aim to show how we managed to treat these rare aneurysms with good outcomes for the patient and microsurgical resolution.

We present a representative case of a 50-year-old female with headache, nausea, language disturbances, left hemiparesis, and deterioration of consciousness, referred to our hospital with a diagnosis of subarachnoid hemorrhage (Hunt & Hess grade 3, Fisher grade 4) secondary to a rupture of a true PComA aneurysm. Urgent surgical clipping was performed, and during surgery, a junctional aneurysm and a true PComA aneurysm were found very close to each other, complicating the procedure, so to have more space to work and perform a safe clipping, we resected the anterior part of the uncus, which broadened the retrocarotid space, performing the clipping with complete exclusion of both aneurysms and no complications. The patient was discharged after five days with right partial III nerve palsy. Postsurgical CT angiography (CTA) and perfusion images showed the permeability of branches distal to the clips.

Knowledge of microsurgical and vascular anatomy is key to the successful treatment of this type of aneurysm, especially when we have multiple aneurysms so close to each other, like in this case, because of the relation with cranial nerves and perforators. Performing extra procedures or transurgical maneuvers like the anterior resection of uncus to broaden our working space is always helpful to avoid complications or major deficits.

## Introduction

Posterior communicating artery (PComA) aneurysms are a group of aneurysms that include three types: 1) “true” PComA aneurysms; 2) junctional aneurysms (in the junction of the internal carotid artery (ICA) and the PComA); 3) aneurysms in the ICA without incorporating the PComA [1, 2]. Posterior communicating artery aneurysms represent 25% of all intracranial aneurysms; however, true PComA aneurysms account for 0.1%-2.8% of all intracranial aneurysms and 6.8% of all PComA aneurysms [3].

Few cases have been reported regarding this type of aneurysm, and around 100 cases were found as true PComA aneurysms in previous literature reviews. An association with other alterations in the vasculature, like vertebral and ICA dissections, PComA fetal type, or hypoplasia, has also been found along these aneurysms. All these findings have been used to demonstrate how flow hemodynamics plays an important role in the physiopathology of true PComA aneurysms [4].

The diagnosis of this aneurysm is a challenge for radiologists and neurosurgeons. The location of these aneurysms, the relationship with the third cranial nerve, and the irrigation and branches of this segment continue to make endovascular treatment difficult, which has given room for clipping and microsurgery as the standard technique of choice [5].

In this case report, we present a patient with a true aneurysm of the PComA and a concomitant junctional PComA aneurysm that was successfully treated by a surgical procedure using, as a helping technique, the resection of the anterior portion of the uncus to broaden the retrocarotid space [6].

## Case presentation

**Figure 1:**
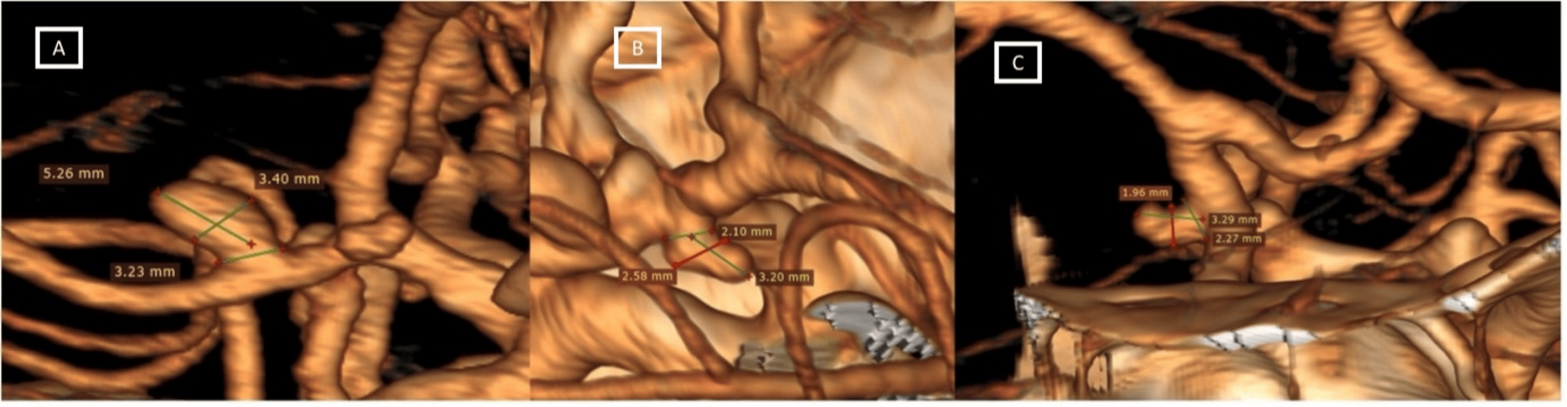
The CT angiography reconstruction shows morphometric characteristics of the aneurysms Pane A represents the true PComA aneurysm. The distance between the true PComA aneurysm and the junction is 2.8 mm. Panes B and C show a junctional PComA aneurysm and its relationship with the surrounding structures. PComA: posterior communicating artery

A 50-year-old female with six hours of thunderclap headache, nausea, dysphasia, left hemiparesis, and deterioration of consciousness arrived at the emergency department. A history of epilepsy treated with phenytoin was noted. On neurological examination, a Hunt Hess grade 3, complete paralysis of the third right nerve, and 4/5 left hemiparesis were identified. A CT angiography (CTA) was performed, and a grade 4 subarachnoid hemorrhage (SAH) in the Fisher classification with an anterior temporal intraparenchymal hemorrhage and two aneurysms were identified. The first aneurysm was a true PComA with a neck of 3.23 mm, a height of 5.26 mm, a dome of 3.40 mm, an aspect ratio of 1.26, and a dome/neck ratio of 1.05. The distance between the junction and the true PComA aneurysm was 2.8 mm (Figure 1). The second one was a junctional PComA with a neck of 2.10 mm, a dome of 2.58 mm, a height of 3.20 mm, an aspect ratio of 1.52, and a dome/neck ratio of 1.22. Urgent surgical treatment was decided.

Surgical technique

**Figure 2:**
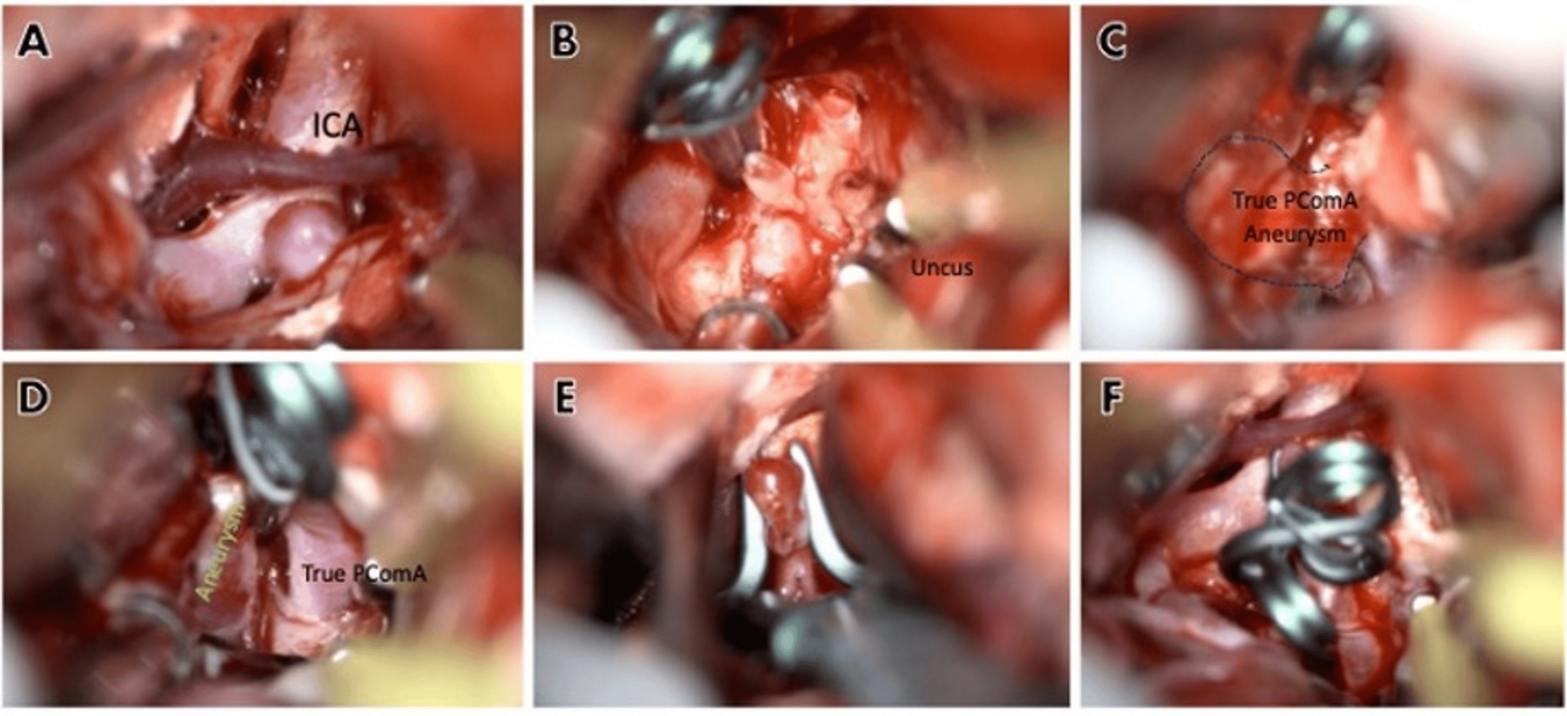
Transurgical images Pane A shows the junctional aneurysm and its relationship with the proximal ICA and PComA segments. Pane B shows a superior view of an image showing a transitional clip on junctional PComA aneurysms, and at the inferior part, we appreciate the partial resection of the uncus. Pane C shows the projection of a true PComA aneurysm and its relationship with surrounding structures. Pane D demonstrates the clipping, or true PComA aneurysm, and its relationship with the PComA. Pane E shows clipping of the junctional aneurysm with a 90 degree-angled clip. Pane F is the final image of both aneurysms clipped. PComA: posterior communicating artery; ICA: internal carotid artery

We used a standard right pterional approach. Microdissection began with the splitting of the Sylvian fissure from anterior to posterior and the opening of the chiasmatic, lamina terminalis, and carotid cisterns. We followed the proximal ICA until the identification of the junctional PComA segment aneurysm, where we put a temporal clip to have control. To have better visualization of PComA and to find the true PComA aneurysm, a partial resection of the anterior portion of the uncus was performed to broaden the retrocarotid space and have a wider working space and maneuverability to avoid damaging surrounding structures. By doing this, we could see the true PComA aneurysm, which had a superiorly projecting dome, so we decided to put a 12 mm straight clip. After this, we removed the temporal clip from the junctional segment aneurysm and changed it to a 5 mm angulated definitive clip (Figure 2). Fluorescein control was performed, showing full closure of both aneurysms and filling of the ICA and anterior choroidal artery (AChoA). Finally, the right mesial temporal hematoma was drained. No complications during surgery were encountered.

**Figure 3:**
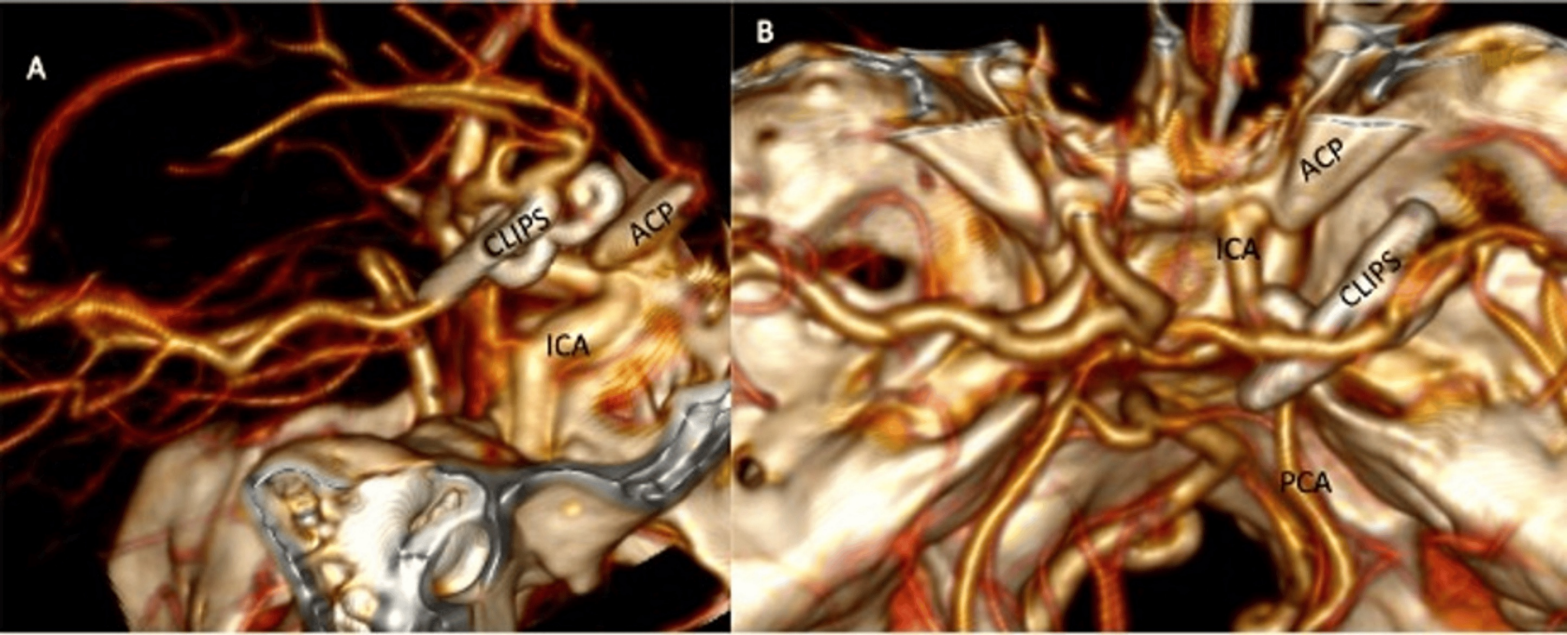
Postoperative control with CT angiography reconstrucción Pane A is a lateral image showing both clips closing the aneurysms. We also appreciate the relationship with the surrounding structures. Pane B is the superior view of the image, showing the relation of the clips with the PCA, with the ICA, and with the ACP. PCA: posterior cerebral artery; ICA: internal carotid artery; ACP: anterior clinoid process

Postsurgical CTA and perfusion images were obtained immediately, showing the permeability of branches distal to the clips with full closure of both aneurysms, without new bleedings, no residual hematoma, and no stroke (Figure 3). The patient stayed 24 hours in intermediate therapy without signs of vasospasm, and then she was admitted to hospitalization, where she stayed for four days. On postsurgical neurological exploration, only partial third nerve palsy was persistent. She was discharged on day five with a Glasgow Coma Scale score of 15 points, right ptosis, and improvement in strength (4/5) on the right side, without added neurological deficits.

## Discussion

True PComA aneurysms were first described by Yoshida in 1979, who called true PComA aneurysms those originating 2-3 mm distal to the junction of the ICA with the PComA [7]. True PComA aneurysms have been described to be located near the ICA, in the middle part of the PComA, or near the posterior cerebral artery [8].

The pathophysiology of aneurysms includes disruption and degeneration of the internal elastic lamina, encompassing hemodynamic changes caused by concomitant alterations in cerebral vascular circulation. Almost all reports of true PComA aneurysms were found in association with added malformations, such as dissections, hypoplasias, or arteriovenous malformations (AVMs) [9].

As in all aneurysms, morphologic characteristics are also important to determine the management. He et al. [10] presented the biomorphometric analysis of true PComA aneurysms to determine if they are more prone to rupture than junctional aneurysms. He reported that PcomA aneurysms are associated with a larger ipsilateral PComA but a smaller P1 segment, which increases fluid dynamics and wall shear stress. No statistically significant difference was found in the size of aneurysms, which was later analyzed by Shin et al. [4], who found that true PComA aneurysms <4mm are more prone to rupture in comparison with those >4mm. This information differs from what was reported by the International Study of Unruptured Intracranial Aneurysms (ISUIA), where aneurysms with a diameter >10 mm were more prone to rupture. This measure changed in the Unruptured Cerebral Aneurysm Study Japan, where a cut-off diameter >7 mm was associated with a higher risk of aneurysm rupture [4].

Diagnosis of true PComA aneurysms continues to be challenging despite the use of digital subtraction angiography (DSA), most of the time being classified as a junctional PComA aneurysm until confirmed transoperative [11]. Accurate diagnosis is key to planning the treatment, including the type of treatment (clipping or endovascular management).

Knowledge of the microsurgical anatomy of the regions is essential to preserving the branches of the PComA [12].

Clinical presentation may include mood changes, memory disturbances, paresis, aphasia, seizures, cranial nerve deficit, and dysmnesia. This may vary depending on the size and dome projection of the aneurysm or if there are other surrounding arteries or branches compromised [13].

Although the pathway to proximal PComA can be achieved with less difficulty through approaches such as a pterional or subtemporal, we must consider other variables such as depth, relationship to adjacent structures like the hypothalamus, mammary bodies, internal capsule, optical and oculomotor nerves, dome projection, and relation with perforators, which may culminate in an unfavorable outcome when clipping. Because of these relations, some authors suggested that treatment options for true PComA aneurysms can be trapping rather than clipping [14, 15].

Surgical treatment is determined by the characteristics of the aneurysm, like dome/neck ratio, dome projection, size, shape, and the relation with surrounding structures. For these aneurysms, one of the variables to be considered is always ensuring the patency of the thalamoperforating arteries, as well as the PComA, the preservation of the oculomotor nerve, the internal carotid, and the optic nerve [16]. To do so, in this area, some surgical techniques or maneuvers can be performed, like, in this case, removing the anterior portion of the uncus to broaden the retrocarotid space and have better visualization of the true PComA aneurysm, avoiding damage to other vascular structures and cranial nerves. Entrapment or trapping should be the treatment of choice for fusiform aneurysms in which clipping is not possible [17, 18].

Although endovascular coiling treatment has obtained favorable results even with an improvement in clinical features unlike clipping, its use is conditioned by the acute angles of the microcatheter required to reach the aneurysm, which can trigger a greater risk of rupture [19]. Therefore, its use is determined for particular cases. In addition to the difference in costs and the ability to resolve any bleeding or hematoma during an imminent rupture, clipping has a certain superiority [20].

## Conclusions

The diagnosis and treatment of true PComA aneurysms are sometimes difficult to make due to the visualization of the vascular complex. Surgical treatment by clipping continues to be a safe and curative option for these aneurysms, but this may be difficult to perform safely when multiple aneurysms in the same segment are present. For this situation, some techniques, like broadening the retrocarotid space by resecting the anterior portion of the uncus to increase maneuverability, can help in the management of this kind of aneurysm, especially in our case where the aneurysm was in close relation to another aneurysm and the risk of rupture and damage to the surrounding structures (like the third nerve or posterior cerebral artery) is higher. We intend to present a representative case of a rare pathology like true PComA aneurysms associated with other aneurysms in the same segment and to highlight the usefulness of concomitant techniques such as resection of the anterior part of the uncus to safely treat this pathology. Successful treatment of this entity requires a good understanding of the microsurgical anatomy and three-dimensional configurations of the aneurysm to preserve all the surrounding neurovascular structures.
